# Unveiling the Effect of Aqueous-Phase Dynamics on Chitosan Hydrogel Film Mechanical Properties Through AFM Nanoindentation and Tensile Testing

**DOI:** 10.3390/gels11070496

**Published:** 2025-06-26

**Authors:** Rafael L. C. G. da Silva, Rômulo Augusto Ando, Denise F. S. Petri

**Affiliations:** Fundamental Chemistry Department, Institute of Chemistry, University of São Paulo, Av. Prof. Lineu Prestes 748, São Paulo 05508-000, Brazil; raando@iq.usp.br

**Keywords:** chitosan swollen films, elastic modulus, AFM nanoindentation, Raman spectroscopy

## Abstract

The mechanical properties of cell scaffolds are strongly influenced by their hydration state. In this study, we investigated the effect of the aqueous phase on the elastic modulus of chitosan hydrogel films using two complementary techniques: uniaxial tensile testing and atomic force microscopy (AFM) nanoindentation. Our results demonstrate that hydration markedly reduced the elastic modulus, decreasing from approximately 2 GPa in dry films to 120 kPa in swollen films, primarily due to the plasticizing effect of water. Moreover, hydrogel films in equilibrium with the aqueous phase exhibited a Young’s modulus three times lower than that of swollen films not in equilibrium. Raman spectroscopy further reveals a solvent “squeeze-out” phenomenon, as evidenced by an increased signal intensity in the 850–1200 cm^−1^ region for stretched films that were out of swelling equilibrium, whereas equilibrated films showed stable spectral features. These findings highlight the crucial role of hydration dynamics in determining the mechanical behavior of chitosan hydrogel films, offering valuable insights for tailoring their properties in biomedical scaffold applications.

## 1. Introduction

Understanding the mechanical properties of biomaterials is essential for their application in fields such as tissue engineering and regenerative medicine. The stiffness of structures (commonly quantified by their elastic moduli) has been a primary focus of research for decades [1,2]. It remains one of the most fundamental parameters, as it can impact the integration of biomaterials within biological systems. The native extracellular matrices might present a wide range of Young’s modulus values, spanning from 1–3 kPa (brain) to 15–40 GPa (bone) [3]. Therefore, modifying the elastic moduli of the cellular environment can affect various aspects of cell function itself, from fundamental processes like adhesion [4,5] and morphology [6,7,8] to more intricate events such as differentiation [9,10,11].

Young’s modulus (E) stands out as one of the most common measurements of intrinsic material stiffness [1]. It is defined as the ratio of applied stress (σ) to the resultant strain (ε) within the linear elastic region of the stress–strain curve. E quantifies the elastic (reversible) deformation of a material when subjected to uniaxial stress, such as tension or compression, making it a fundamental property for characterizing mechanical behavior.

The determined values of the mechanical properties can vary depending on the method used and whether the sample is dry or wet. Uniaxial tensile testing is a macroscopic method commonly used to evaluate the E of bulk samples [1,12]. It is advantageous for measuring the global mechanical properties of homogeneous materials and assessing their large-scale structural integrity [12]. When it comes to biomaterials, their inherent variability in composition and structure can lead to overlooked localized variations in stiffness, resulting in measurements that do not accurately reflect their overall mechanical behavior [13].

The scale dependency of mechanical properties poses an additional challenge for uniaxial tensile testing experiments [14]. While it provides a macroscopic measurement, it fails to capture critical nanoscale and microscale interactions, such as those between cells and the extracellular matrix, which are crucial for understanding the performance of biomaterials in biological environments [15]. Additionally, factors like hydration [16,17,18] and temperature [19,20] significantly influence mechanical properties, but these are difficult to control in macroscopic testing, limiting the reproducibility and relevance of the results for in vivo conditions [21].

Alternative techniques have gained prominence due to their ability to address limitations associated with tensile testing. Non-invasive methods such as magnetic resonance elastography [22] and ultrasonic shear wave elastography [23] enable in vivo measurements of soft tissue stiffness under physiological conditions, providing valuable insights into the dynamic mechanical behavior of living tissues. Micropipette aspiration [24], which measures the deformation of tissues or cells under applied pressure, offers a versatile approach for characterizing the mechanical properties of soft biomaterials at the microscale.

Probe indentation techniques provide a robust method for measuring localized stiffness in heterogeneous materials [24]. In this case, deformations are maximized at the point of indenter contact and radially diminish to zero with increasing distance from the probe [12]. The nanoindentation technique is a smaller-scale version of the engineering indentation method of hardness testing. Because it was designed for analysis of stiff and hard surfaces (like metals and silicon), nanoindentation was promptly adapted to the study of mineralized biological materials, such as bone and teeth [25,26]. More recently, nanoindentation has also been applied to the study of soft and hydrated biomaterials such as hydrogels [27].

AFM indentation involves a cantilever with a sharp tip, or colloidal probe, which approaches the sample surface under precise control. As the probe makes contact with the surface, it applies a controlled force, and the resulting indentation depth is measured. The interaction between the probe and the sample generates a force–displacement curve, also known as a force curve, which is recorded in real time. This curve consists of a loading phase, where the probe indents the material, and an unloading phase, as the probe retracts. The resulting force (F) is dependent on the indenter geometry and the indentation depth (δ), as described in Equation (1). For a sphere indenting a flat surface to an indentation depth of δ:

(1) $$F(\delta )\;=\;\frac{4}{3}\;\frac{E\sqrt{R}}{\left(1-v^{2}\right)}\delta^{3/2}$$

where E represents the Young’s modulus of the sample, R the colloidal probe radius, and ν the Poisson’s ratio of the sample [12]. For hydrogels, ν is typically 0.5 [28]. It is important to note that this approach is only valid for small, purely linear elastic indentation depths, where δ ≪ R.

Despite its high force resolution, AFM nanoindentation faces some drawbacks. Data acquisition and analysis are often time-consuming and technically demanding, requiring extensive calibration and expertise. Furthermore, measurements are restricted to small sample areas and can be affected by surface roughness or heterogeneity, potentially misrepresenting the material’s bulk properties. These limitations collectively reduce its practicality for routine or large-scale analyses [28,29].

Disparities in E values obtained from local and bulk measurements reflect their inherent differences in scale and the specific mechanical responses they capture. Tensile testing, which involves bulk deformation, tends to yield higher E values. These bulk properties often dominate the mechanical response, masking the contributions of localized features. Conversely, indentation tests isolate the mechanical properties of small-scale regions, highlighting the influence of microstructural heterogeneities. Table 1 presents the E values calculated using various testing methods for biomacromolecule-based scaffolds in both dry and wet states, processed in different forms.

Chitosan (CHI) films are commonly used in both cell culture and in vivo implantation, where they are exposed to aqueous environments such as culture media or tissue fluids. In these conditions, hydration significantly alters their mechanical properties, particularly stiffness (E), which, in turn, affects cell behavior and scaffold performance [46,47,48,49]. Understanding how E changes across hydration states is, therefore, essential for designing chitosan-based materials that function effectively under physiological conditions and exhibit stiffness levels compatible with the mechanical requirements of specific cell types.

Electro-spun CHI scaffolds reinforced with polycaprolactone (PCL) and cross-linked with genipin enhanced mechanical stability and biocompatibility by 63% compared to pure CHI. Cross-linked CHI/PCL scaffolds with ultimate stress of 5.4 ± 1.1 MPa showed slower degradation and supported higher cell viability, while non-cross-linked samples degraded rapidly and released toxic residues, reducing viability [50]. More recently, Rosova et al. [47] investigated chitosan–polyacrylamide hydrogels and evaluated their mechanical behavior under different swelling conditions, revealing a strong influence of pH and hydration on the swelling degree and mechanical properties, with tensile moduli around 37 kPa in the swollen state. Chitosan–PDMS hybrid scaffolds with tunable stiffness showed that scaffold mechanics directly influenced C2C12 myoblast behavior. Scaffolds with softer properties (~62 kPa) resulted in poor adhesion and clustered cells. In contrast, scaffolds with intermediate stiffness (114–568 kPa) promoted better cell viability, spreading, and an elongated morphology, which are typical features of healthy myoblasts. In contrast, the stiffest scaffolds (~691 kPa) altered cell shape, suggesting that excessive stiffness can impair cell organization [36]. While mechanical properties were also measured under physiological conditions, the role of hydration state itself and the potential influence of testing methodology on measured E values were not thoroughly addressed.

Despite these recent findings, the literature lacks studies that systematically investigate how E changes across hydration states, or how different mechanical characterization methods capture these variations for chitosan materials. This work focused on analyzing, in detail, how the hydration state of chitosan hydrogel films affected their mechanical response. Using both uniaxial tensile testing and AFM nanoindentation, we systematically compared the E values of dry, swollen (out of equilibrium), and fully hydrated (equilibrated) films. We further employed Raman spectroscopy to explore water dynamics within the films and correlated these findings with mechanical behavior.

## 2. Results and Discussion

### 2.1. Microstructure of Cast-Dried CHI Films

**Figure 1:**
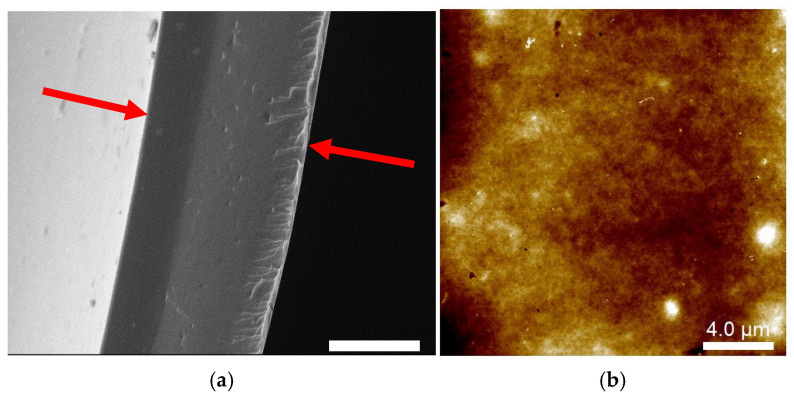
(**a**) SEM image of the microstructure of cast-dried chitosan film cryofracture in liquid N_2_. The scale bar corresponds to 50 µm. The film thickness (indicated by the red arrows) was 90 µm. (**b**) AFM topographic image of dry CHI film surface.

The microstructure of cast-dried chitosan films was analyzed using scanning electron microscopy (SEM) and atomic force microscopy (AFM), as shown in Figure 1. The micrograph of a CHI film cross-section in Figure 1a highlights a dense polymeric matrix with an apparent regular and smooth surface (with small irregularities). A similar film structure was previously reported by Breda et al. [34]. The dry films had an average thickness of (96 ± 11) µm, consistent with the thickness observed in the SEM image (indicated between the red arrows).

The AFM image in Figure 1b confirms the presence of localized, nanometric, surface globular-like structures (bright spots). Such domains have recently been attributed to the intrinsic self-assembly behavior of chitosan chains driven by hydrogen bonding and electrostatic interactions during the film formation process [51,52,53]. Additionally, residual N-acetyl groups along the chitosan chains would also lead to aggregation due to their poor solubility in acidic media [52]. Surface cavities are also in the nanometric range (Figure S1), as previously reported by Assis et al. [53], which is in agreement with a collapsed film structure formed during the solvent casting process.

### 2.2. Aqueous-Phase Sorption Studies

**Figure 2:**
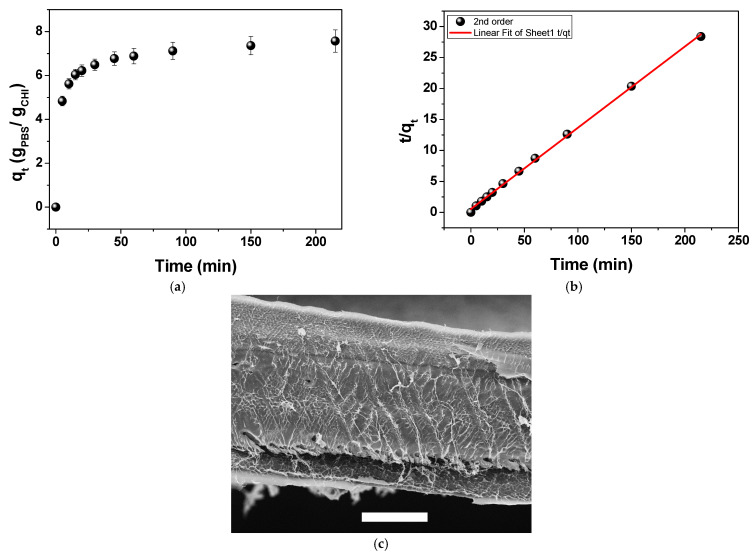
(**a**) Mean values of PBS absorbed amount (q_t_) as a function of time performed on CHI films (triplicate). (**b**) Linear fitting to the pseudo-second order equation, y = 0.5371 + 0.13125x, R^2^ = 0.9990. (**c**) SEM image of the cryofracture freeze-dried CHI film in liquid N_2_. The scale bar corresponds to 50 µm.

Figure 2a shows the sorption kinetics of the CHI film in PBS. The amounts of PBS uptake per mass of CHI at equilibrium and at any time t (min) are denoted as q_e_ and q_t_, respectively. The sorption kinetics behavior can be quantitatively evaluated using the pseudo-first order and pseudo-second order Equations (2) and (3), respectively:

(2) $$\ln\left(q_{e}-q_{t}\right)=-k_{1}t+lnq_{e}$$

(3) $$\frac{t}{q_{t}}=\left(\frac{1}{q_{e}}\right)t+\frac{1}{k_{2}q_{e}^{2}}$$

where k_1_ and k_2_ are sorption rate constants.

The experimental sorption data fitted the second-order model (Figure 2b) better than the first-order model (Figure S2), indicating that PBS progressively wetted the polymer chains rather than instantaneously, and that the sorption rate depends on the square of the available surface area. From the fitting parameters, q_e_ and k_2_ were determined to be 7.62 g PBS/g CHI and 0.032 g CHI/g PBS, respectively. Figure 2a shows that the sorption is fast up to 20 min, achieving approximately 80% of the equilibrium with the aqueous phase. This rapid initial sorption phase might be attributed to the immediate hydration of the film’s outer polymer network. The subsequent slower phase reflects the diffusion-limited penetration of PBS into the inner regions of the film, governed by their collapsed pore structure observed in Figure 2c. The dense morphology of an interconnected fibrillar structure [54] probably hinders PBS from promptly penetrating deeper layers of the film, prolonging the hydration.

### 2.3. Film Mechanical Characterization

#### 2.3.1. Tensile Testing

**Figure 3:**
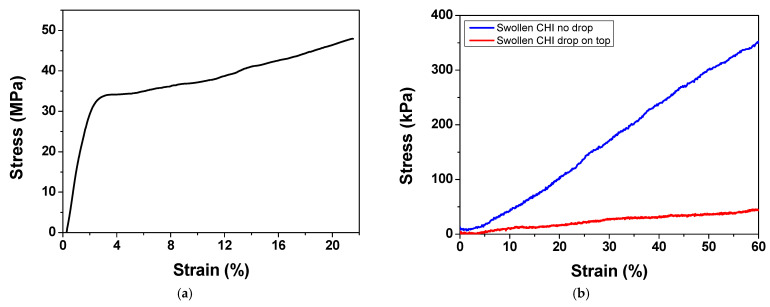
Representative stress–strain curves obtained for (**a**) dry CHI films or (**b**) PBS-swollen CHI films without (blue line) or with (red line) a PBS drop on the surface. For all sets of tensile experiments, tensile tests were performed on ten samples, consisting of five specimens from each of two independent batches.

Figure 3a shows a typical stress–strain curve for dry CHI films. They presented a strain-hardening behavior. On the other hand, PBS-swollen CHI films exhibited a hyper-elastic nature (Figure 3b). The hyper-elastic behavior of hydrated collagen films has been reported and attributed to the sliding of the collagen fibrils, thanks to the presence of water molecules [30,32,55,56]. Stress–strain curves for swollen CHI films exhibited a strain-stiffening behavior, also reported for soft collagenous tissues [30,57] and referred to as the J stress–strain curve. Initially, the polymer chains within the hydrated network remain more disordered. For values up to 5% strain, there is a slower increase in stress (Figure 3b). As the polymer chains align, under constant stress, hydrogen bonding and chain entanglements increase, enhancing the stiffness. At higher strain levels, chain slippage and delamination occur, eventually leading to fracture. Genipin-cross-linked chitosan–PCL electro-spun scaffolds also displayed J-shaped stress–strain curves and strain-stiffening behavior [50].

While strain-stiffening behavior is typically associated with fibrous proteins such as collagen, recent studies have shown that chitosan-based hydrogels can also exhibit nonlinear mechanical responses under deformation. Liu et al. demonstrated biomimetic strain-stiffening in O-carboxymethyl chitosan hydrogels cross-linked via dynamic imine bonds, where the differential modulus increased with applied strain, mimicking the behavior of the extracellular matrix (ECM) [58]. Lee et al. observed nonlinear stress–strain behavior in porous poly(vinyl alcohol)–chitosan hydrogels under unconfined compression, which they modeled using an exponential function commonly associated with strain-stiffening behavior in soft biomaterials [48]. These effects were more pronounced in swollen films without PBS on their surface (blue line). In contrast, films with PBS on their surface (red line) showed only a slight increase in stiffness under stress, likely due to weak chain alignment. In this case, the buffer droplet maintains local hydration, preventing solvent expulsion and thereby reducing polymer chain densification, while limiting orientation along the stretching direction. Figure S3 shows the distribution of E for CHI films in the swollen state, obtained under the strain rates of 1.0 and 1.5 mm/min, via tensile testing. While, on average, E values were higher at the 1.5 mm/min strain rate, statistical analysis revealed no significant differences between the two tested conditions. This indicates that, within this range, the mechanical behavior of the films is not substantially affected by the applied strain rate.

#### 2.3.2. AFM Nanoindentation

**Figure 4:**
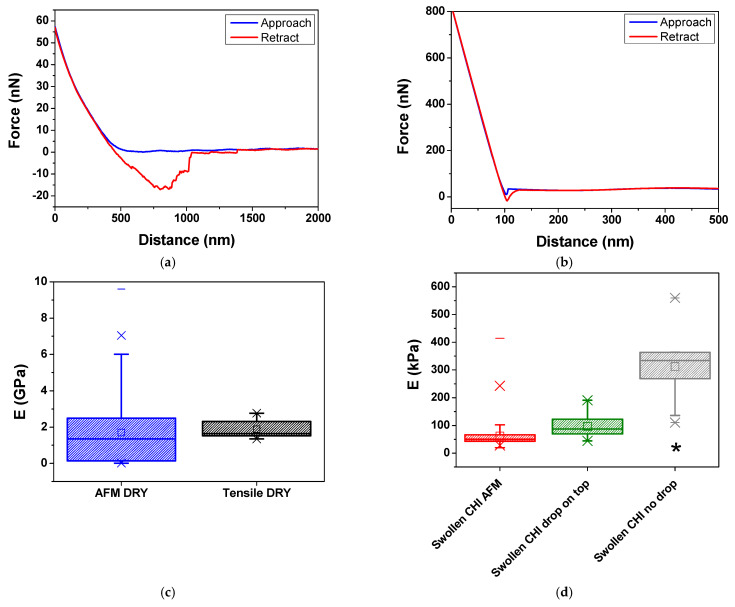
Representative force curves obtained for (**a**) PBS-swollen and (**b**) dry CHI films using 10 μm nominal diameter colloidal silica spheres. Distributions of E values determined for CHI films in the (**c**) dry state and (**d**) swollen state obtained via tensile testing or AFM nanoindentation. Mean values (■), minimum and maximum values (−), mean 1% and 99% values (×), and median values (―). * *p* < 0.05. For AFM nanoindentation experiments, three random regions from two samples of different batches were analyzed, resulting in a total of 1000 force curves. Tensile tests were performed on ten samples, consisting of five specimens from each of two independent batches.

Figure 4a,b presents typical AFM force volume curves for dry and PBS-swollen CHI films. Figure 4c,d compares the distributions of calculated E using tensile testing and force volume curves for dry and swollen CHI films, respectively. The distribution of E values from Figure 4c,d is also presented as histograms for the AFM force–volume of dry and swollen CHI films in Figure S4.

For dry CHI films, the tensile tests yielded E values in the range of 1.5 to 2.8 GPa, while the AFM force volume measurements exhibited a significantly broader range, spanning from 10 MPa to 9.6 GPa (Figure 4c). This significant difference highlights the inherent scale-dependent nature of both methods. Tensile testing measures the bulk mechanical properties of the entire film under macroscopic deformation, capturing the collective response of the polymer network [1,12]. In contrast, AFM indentation probes localized mechanical properties at the nanoscale [29], where chitosan’s intrinsic heterogeneity may lead to substantially higher modulus values. Nevertheless, both techniques present a mean value of 2 GPa for E, which is in agreement with results previously reported in the literature for dry chitosan films [33,34,35,39].

Dry CHI films presented substantially higher E values than the PBS-swollen CHI films. Tensile tests conducted on swollen films out of equilibrium (without a PBS drop on the surface) yielded a mean E value of 290 kPa. In contrast, when the swollen films were maintained in equilibrium, either through AFM nanoindentation or tensile tests with a PBS drop on the surface, the mean E values were lower, ranging from 60 to 100 kPa, as presented in Figure 4d. The continuous presence of PBS during these tests kept the polymer chains highly solvated, allowing for greater mobility and elongation, thereby enhancing the plasticizing effect of the films [32,55,56]. On the other hand, in the absence of a PBS drop on the surface, the polymer chains elongated under stress, expelling part of the solvent and reducing the free volume between them. As a result, the films became stiffer.

The relaxation effect of the CHI chains is evident in the mechanical properties of the film, as demonstrated by the significant reduction in elastic modulus when comparing dry films to hydrated ones (from GPa to kPa), for both techniques. In dry films, the elastic modulus is higher due to the absence of buffer, which results in a stiffer and more densely interconnected polymer network, as previously observed via SEM micrography.

We hypothesize that differences in observed values of E could be explained by the aqueous phase dynamics in the different systems. The absence of an additional buffer droplet on the film surface during tensile testing of swollen films would promote greater expulsion of the aqueous phase contained within the polymer matrix as the material undergoes deformation (thickness reduction along the z-axis) and drying. This phenomenon reduces the amount of buffer retained within the film, resulting in a stiffer and less plasticized polymer matrix, which leads to higher E values. Conversely, in films where sorption equilibrium is maintained, the greater presence of liquid at the interface may mitigate this expulsion effect, allowing the material to remain hydrated throughout the tensile test. This contributes to the observed lower E values, maintaining the system’s flexibility. This would also explain why the calculated E values are not statistically different from the ones obtained via the AFM technique (*p* < 0.05).

It is essential to note that measurements of mechanical properties via AFM nanoindentation are also susceptible to dynamic effects in the aqueous phase. In this case, one should take into account confinement effects [27,59]. Under unconfined conditions, the hydrogel layer can freely expand, allowing liquids to flow in multiple directions without significant external constraints. This facilitates aqueous-phase migration away from the indented region, leading to greater fluid displacement and faster pressure equilibration within the hydrogel. A confined condition would restrict fluid migration. This confinement results in one-dimensional radial flow patterns and increased pressure buildup beneath the indenter, which affects the apparent stiffness of the hydrogel. For this reason, indentations were performed to a much smaller depth compared to the swollen film thickness. From Figure 4a, a typical indentation for swollen CHI was about 0.5 mm, whereas the average thickness for swollen CHI films was around 400 μm, as presented in Table S1.

While AFM nanoindentation and uniaxial tensile testing inherently probe distinct mechanical modes and operate at different scales, our primary objective in employing both methods was to comprehensively examine how hydration state influences the material’s mechanical response. This dual-scale approach allowed us to elucidate how solvent-mediated effects, such as plasticization and solvent expulsion, manifest differently from the micro- to macro-scales.

### 2.4. Aqueous-Phase Dynamics Studies

#### 2.4.1. Raman Spectroscopy

**Figure 5:**
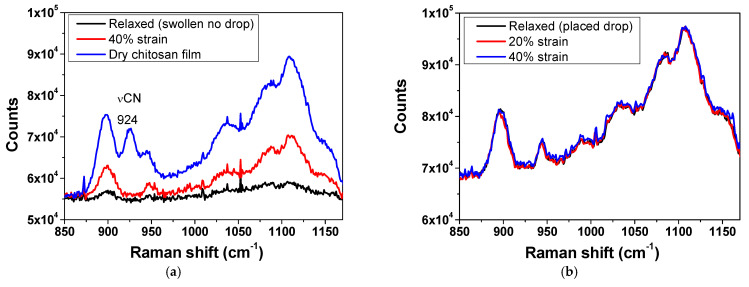
Raman spectra obtained for CHI films (duplicate) in a relaxed state and under stress: (**a**) dry or swollen CHI films without PBS drop, and (**b**) swollen CHI films with PBS drop on the surface of the film.

To qualitatively assess aqueous-phase dynamics within the CHI hydrogel film matrix, Raman spectroscopy was employed. Raman spectroscopy has been previously utilized on porcine skin samples to monitor spectral variations associated with water content [60]. Figure 5 presents Raman spectra for swollen CHI films, comparing conditions with and without a PBS drop on the surface, both in the absence of stress and under stress.

No spectral shift could be observed after CHI hydration or when the swollen films were under stress. The main difference observed was the suppression of the band at 924 cm^−1^, assigned to the C-N stretching mode, ν (CN) [61]. This suppression is probably related to conformational changes of chitosan chains when swollen [61]. For the spectrum of the swollen film without the PBS droplet (Figure 5a), the Raman signal intensity progressively increased with the applied tensile stress, which was also observed for porcine skin samples under different hydration states [60]. It is essential to mention that besides the scattering properties of the sample, the intensity of Raman scattered light depends on the concentration of the Raman-active molecules within the probe volume illuminated by the excitation laser [62].

This phenomenon can be correlated with the “solvent squeeze-out” effect, where water is expelled from the polymer matrix during deformation, resulting in sample compaction. A similar effect was reported for swollen collagen films when submitted to tensile tests under buffer or in air [32]. Simic et al. also reported the increase in the concentration of polymer in the near-surface region of PAAm hydrogels during compression, as evidenced by time-resolved ATR-FTIR spectroscopy, indicating solvent exudation from the brushy hydrogel surface [63]. As previously discussed, Figure 2a shows a rapid sorption, which is related to the solvation of the outer layers. It is likely that the solvent molecules present in the outer layers of the film have higher mobility, making their release easier. The solvent expulsion leads to an increase in the effective “concentration” of chitosan within the film, resulting in a higher density of polymer chains in the analyzed region and, consequently, an increase in the Raman signal intensity. In contrast, for the spectrum of the film in equilibrium with the aqueous phase (Figure 5b), no significant variation in Raman signal intensity was observed as the tensile stress increased. The presence of the PBS droplet on the surface appears to mitigate the “squeeze-out” effect, maintaining the film’s hydration and preventing local concentration of chitosan. This suggests that the additional droplet acts as a solvent reservoir, allowing the material’s solvent balance to be maintained during the tensile process, which results in the stability of the Raman signal intensity.

#### 2.4.2. Gravimetrical Analysis

Solvent loss was observed when gravimetric analysis was performed on swollen CHI films, both those subjected to and those not subjected to tensile experiments (Table S2, five replicates), at 20°C and 60% relative humidity. CHI films, when subjected to uniaxial tensile tests, exhibited a greater loss of the aqueous phase compared to their non-stretched controls. On average, stretched films lost 14 ± 5% of their initial mass, while control films exhibited a loss of only 4 ± 1%. This difference was statistically significant, indicating that the film’s mechanical deformation facilitates the release of aqueous material from within the polymeric matrix. The applied strain likely expels loosely bound water faster, leading to increased dehydration of the material.

Another factor supporting the hypothesis of the water “squeeze-out” effect in swollen chitosan films relates to their free water population. Previously, we used time-domain nuclear magnetic resonance (TD-NMR) to quantify the relaxation times (T2) of hydrogen atoms in different chitosan hydrogels after cross-linking with vanillin [35]. In this technique, signal intensity is proportional to the number of hydrogen nuclei in water molecules within each pore population. At the same time, the decay rate (or T2 time) corresponds to the mobility of water molecules in those pores [64].

Water confined in small pores exhibits short relaxation times, whereas water in larger pores corresponds to longer relaxation times. The longest relaxation times observed represent the free water population. Bulk water has T2 values exceeding 2000 ms, but these values decrease in swollen polymer matrices due to water–polymer interactions [65]. For chitosan, T2 values around 115 ms account for 98.8% of the water in the film, while another band at 14 ms was barely detected. This indicates that most of the water sorbed by the film is weakly bound to the polymer matrix and would easily leave the material, especially when under external stress.

## 3. Conclusions

This study highlights the critical role of hydration state and measurement methodology in determining the mechanical properties of CHI films, addressing a key gap in the literature regarding E values across different hydration conditions. By systematically comparing uniaxial tensile testing and AFM nanoindentation, we demonstrate that the elastic modulus of chitosan films spans from the GPa range in dry conditions to the kPa range in swollen states, emphasizing the plasticizing effect of water due to CHI chain relaxation. Our results reveal that uniaxial tensile testing generally yields higher E values than nanoindentation, particularly when solvent expulsion occurs during deformation. However, when hydration is maintained, either through nanoindentation in an aqueous medium or by introducing a buffer droplet during tensile testing, E values become comparable between the two methods. This highlights the influence of hydration equilibrium on mechanical measurements, underscoring the importance of physiologically relevant testing conditions. Furthermore, Raman spectroscopy confirms that solvent expulsion during tensile testing increases local polymer concentration, directly impacting mechanical behavior. This solvent redistribution effect provides a mechanistic explanation for the observed variations in E and further supports the importance of hydration control in biomaterial characterization. By bridging the gap between conventional mechanical testing in dry conditions and the realistic hydrated environment of biomaterials, our findings contribute to a more accurate assessment of chitosan films for biomedical applications. Since both cell culture and in vivo implantation involve aqueous environments, characterizing scaffolds in their entirely or partially hydrated state is crucial for predicting the actual mechanical environment that cells and tissues experience. The insights gained here are expected to improve the design and optimization of chitosan-based scaffolds for tissue engineering and regenerative medicine, where mechanical properties must be carefully tailored to mimic physiological conditions.

## 4. Materials and Methods

### 4.1. Chitosan Film Preparation

High-molecular-weight chitosan (CHI, Sigma-Aldrich 419419, St. Louis, MO, USA) was used without further purification. M_v_ was 859 kg/mol, and DD = 70% [36]. Firstly, stock solutions of CHI (10 g/L) were prepared by dispersing the polysaccharide in a 2% acetic acid solution under magnetic stirring, at room temperature, for 24 h. Insoluble impurities were removed by centrifuging the previously dispersed CHI in 15 mL vials at 3600 rpm for 45 min. Afterwards, the supernatant was transferred to a new flask and magnetically stirred for 1 h to avoid any gradient in molar mass or CHI concentration that might have occurred due to the centrifugation step. Films were produced at room temperature by casting the solution onto plastic petri dishes with a 3.0 cm radius and a 1.5 cm height.

### 4.2. Sorption Kinetics

The sorption kinetics of the films in phosphate buffer saline (PBS, 137 mM NaCl, 10 mM phosphate, 2.7 mM KCl, pH 7.4) was determined gravimetrically at (24 ± 1) °C and 60% relative humidity. Dried CHI films were cut into pieces (1 cm × 1 cm) and weighed with an accuracy of 0.1 mg to determine their dry mass, m_CHI_. Then, the films were completely immersed in PBS. After 5, 10, 15, 30, 45, 60, 90, 150, and 215 min, they were removed from the PBS, and the excess liquid was carefully removed using filter paper. The swollen films were then weighed to determine their swollen mass, m_swollen_. The difference between m_swollen_ and m_CHI_ results in m_PBS_. PBS was chosen because it provides the same pH and ionic strength as those used in the cell culture experiments. The amount of PBS sorbed per dry CHI film at a given time t, qt, (g_PBS_/g_CHI_), was determined for triplicate samples by Equation (4):

(4) $$q_{t}=\frac{m_{PBS}}{m_{CHI}}$$

### 4.3. Macro Mechanical Testing

**Figure 6:**
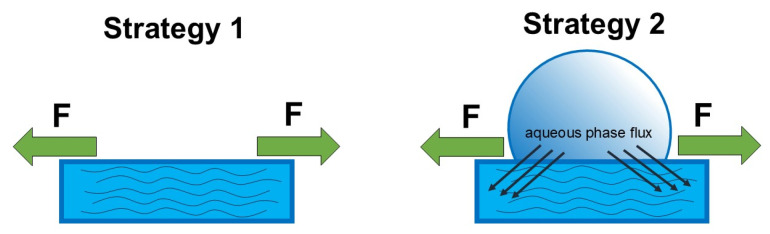
Scheme representing both strategies applied in the uniaxial tensile experiments for swollen CHI films.

Uniaxial tensile experiments were performed using a Deben^®^ Microtest 200N (Suffolk, UK) (Figure S1). For that, dry films were cut into rectangular-shaped pieces (0.5 cm × 2 cm). Film thickness was accessed using a Digimess micrometer (São Paulo, Brazil) (0.01 mm precision) in the dry and swollen states at three different regions. For swollen films, two strategies were used: (1) films equilibrated in buffer were blotted dry before the experiment, and (2) films were directly mounted with a 100 µL droplet covering the whole film surface. This volume was chosen as it was sufficient to cover the central gauge region without spilling beyond the film area or interfering with the grips. This approach allowed us to assess the role of local hydration equilibrium in mechanical response, without immersing the entire film during testing. Strategy 2 aimed to maintain the sorption equilibrium between the aqueous phase and the film throughout the tensile tests, as shown in Figure 6.

Tensile tests were performed on ten samples, five specimens from two independent batches, at strain rates of 1.0 and 1.5 mm/min. These rates were selected to minimize water evaporation, as no buffer drop was applied to the swollen films during the testing process. Each test took either 10 min (at 1.0 mm/min) or 6.7 min (at 1.5 mm/min). The experimental setup is shown in Figure S5.

### 4.4. Micro Mechanical Testing

The elastic modulus (E) of the films was measured using a Bruker Multimode-8 AFM (Bilerica, MA, USA) operating in force spectroscopy mode. Colloidal silica spheres (10 μm nominal diameter) were used as indenting probes, fixed with epoxy glue to the end of tipless cantilevers with a spring constant of 8.9 N/m (MikroMasch HQ: NSC35/tipless/No Al, Sofia, Bulgaria), as shown in Figure S6. The films were measured in the dry state or swollen in PBS for at least 24 h, respecting their average swelling equilibrium time. In the case of swollen CHI films, force–volume measurements were performed using the colloidal probe, with the hydrogel film completely immersed in the aqueous medium to maintain the film swollen and in equilibrium with the buffer aqueous phase during the measurements. Then, 16 × 16-pixel maps were recorded at three random regions of two samples from different batches. A total of 500 force curves were collected for each sample.

### 4.5. Spectroscopic Characterization

Raman spectra were acquired using a Renishaw inVia-Reflex spectrometer (Wotton-under-Edge, UK) with a 785 nm excitation laser, covering the spectral range of 850–1200 cm^−1^, and a 20× objective. CHI films were analyzed under three conditions: unstrained and subjected to uniaxial strain of 20% and 40%. The films were examined in the dry state, in a swollen state without a PBS droplet, and a swollen state with a PBS droplet on the surface. Averaged Raman spectra are available in Figure S7.

### 4.6. Aqueous-Phase Loss Evaluation

Aqueous-phase loss from CHI films was gravimetrically evaluated in a Mettler Toledo AX205DR Delta range Analytical Semi-Micro Balance (0.01 mg precision). Firstly, CHI films were equilibrated in phosphate-buffered saline (PBS, pH 7.4) for 24 h to allow maximum aqueous-phase uptake. After equilibration, the films were removed from the solution, and the excess surface buffer was blotted using lint-free tissue paper. Each experimental run included a pair of CHI films, where one was subjected to uniaxial tensile testing (the sample), while the other remained untreated (the control). Five repetitions were performed, as provided in Table S2. Immediately before and after the tensile test, both films were weighed to follow mass loss. The percentage of aqueous-phase loss was calculated by comparing the initial and final masses of each film.

### 4.7. Statistical Analysis

One-way analysis of variance (ANOVA) was performed, with a significance level of *p* < 0.05. Experimental data were reported as the interquartile range (IQR), which represents the middle 50% of the dataset, minimizing the influence of outliers. The first quartile (Q1) and third quartile (Q3) define the lower and upper bounds of the interquartile range (IQR), respectively, providing a robust measure of data dispersion.

## Figures and Tables

**Table 1:** Elastic modulus (E) values determined using various testing methods for biomacromolecule-based scaffolds in both dry and wet states, processed in different forms.

System	Form	E (GPa)		Testing Method	Ref.
		Wet	Dry		
Collagen	Film	0.006–0.009	8.5 ± 4.2	Nanoindentation	[30]
	Fibril	0.0012 ± 0.0001	1.9 ± 0.5	AFM	[31]
	Film	0.00274 ± 0.001	0.0757 ± 0.0157	Tensile testing	[32]
Chitosan	Film	-	1.5	Nanoindentation	[33]
	Film	-	0.1344 ± 0.0174	Tensile testing	[34]
	Film	-	0.236 ± 0.027	Tensile testing	[35]
	Film	0.000137 0.000062	0.275 -	Tensile testing Nanoindentation	[36]
	Film	0.00348 ± 0.00024	-	Tensile testing	[37]
	Thin film	0.3–0.7		AFM	[38]
	Film	-	1–3	AFM	[39]
	Hydrogel	0.00006542	-	Compression	[40]
Sodium Alginate	Film	-	0.0129 ± 0.0012	Tensile testing	[41]
	Hydrogel	0.000015–0.000045	-	AFM	[42]
	3D-printed	-	-	AFM	[43]
	hydrogel	0.00015–0.00055	-	Nanoindentation	[44]
	film		0.0456 ± 0.0041	Tensile testing	[45]

## Data Availability

The original contributions presented in this study are included in the article/Supplementary Materials. For further inquiries, please contact the corresponding authors.

## Supplementary Materials

The following supporting information can be downloaded at: https://www.mdpi.com/article/10.3390/gels11070496/s1, Figure S1: (a) Zoomed in AFM image of dry CHI film surface. (b) Film cross-section highlighting the presence of a nanometric surface pore; Figure S2: Linear fitting to the pseudo-first order equation, y = 0.71074 − 0.01628 x, R^2^ = 0.93447; Figure S3: Distributions of E for CHI films in the swollen state obtained via tensile testing at different strain rates. Mean values (■), minimum and maximum values (−), mean 1% and 99% values (×), and median values (―). * *p* < 0.05; Figure S4: Histograms obtained for the indentation of CHI films; 16 × 16-pixel maps were recorded at three random regions of two samples from different batches. A total of 500 force-curves were collected for each sample; Table S1: Average film thickness accessed using a Digimess micrometer (0.01 mm precision) in the dry and swollen states, under different conditions; Table S2: Changes in the mass of swollen CHI films subjected to tensile testing (sample) and not tested (control). The time point t_0_ indicates when the films were removed from PBS and blotted dry. The time point t corresponds to the moment after the tensile test when the mass of both the sample and control films was measured. The normalization factor was obtained from the highest and lowest film mass ratios; Figure S5 shows a swollen CHI film with a buffer drop placed on top. Tensile tests were performed with a Deben^®^ Microtest 200N, at a constant rate of 1.5 mm/min and an initial gap of 10 mm between the clamps; Figure S6: Optical microscopy of a colloidal silica sphere fixed with epoxy glue to the end of an 8.9 N/m tipless cantilever. The scale bar corresponds to 20 mm; Figure S7: Raman spectra obtained for CHI films in a relaxed state and under stress: (a) dry or swollen CHI films without PBS drop, and (b) swollen CHI films with PBS drop on the surface of the film. Spectra were averaged for n = 2.
