# Supplementary material for: Unveiling the Effect of Aqueous-Phase Dynamics on Chitosan Hydrogel Film Mechanical Properties Through AFM Nanoindentation and Tensile Testing
Source: Gels. 2025 Jun 26;11(7):496. doi: 10.3390/gels11070496 (PMC12296060; doi:10.3390/gels11070496)
Supplement: Supplementary file 1 [file gels-11-00496-s001.zip › gels-3703425-supplementary.pdf]

Supporting information

Unveiling the effect of aqueous phase dynamics on chitosan hydrogel film mechanical properties through AFM nanoindentation and tensile testing

Rafael L. C. G. da Silva [1,*], Rômulo A. Ando [1] and Denise F. S. Petri [1,*]

[1] Fundamental Chemistry Department, Institute of Chemistry, University of São Paulo, Av. Prof. Lineu Prestes 748, 05508-000, São Paulo, Brazil;

* Correspondence: rafaelcruz@iq.usp.br (RS); dfsp@iq.usp.br (DP)

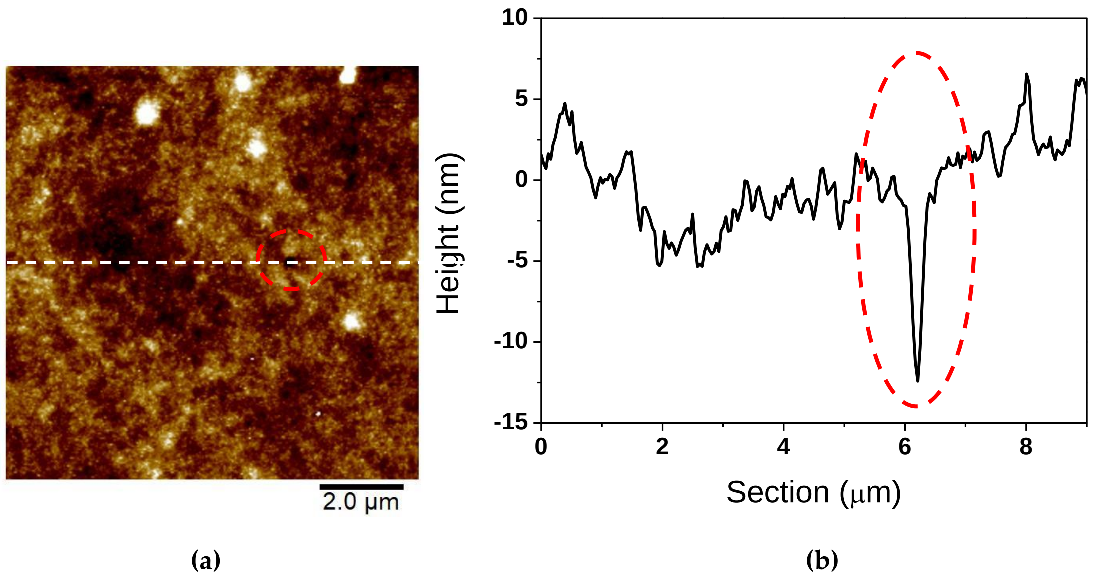

Figure S1. (a) Zoom in AFM image of dry CHI film surface. (b) Film cross-section highlighting the presence of a nanometric surface pore.

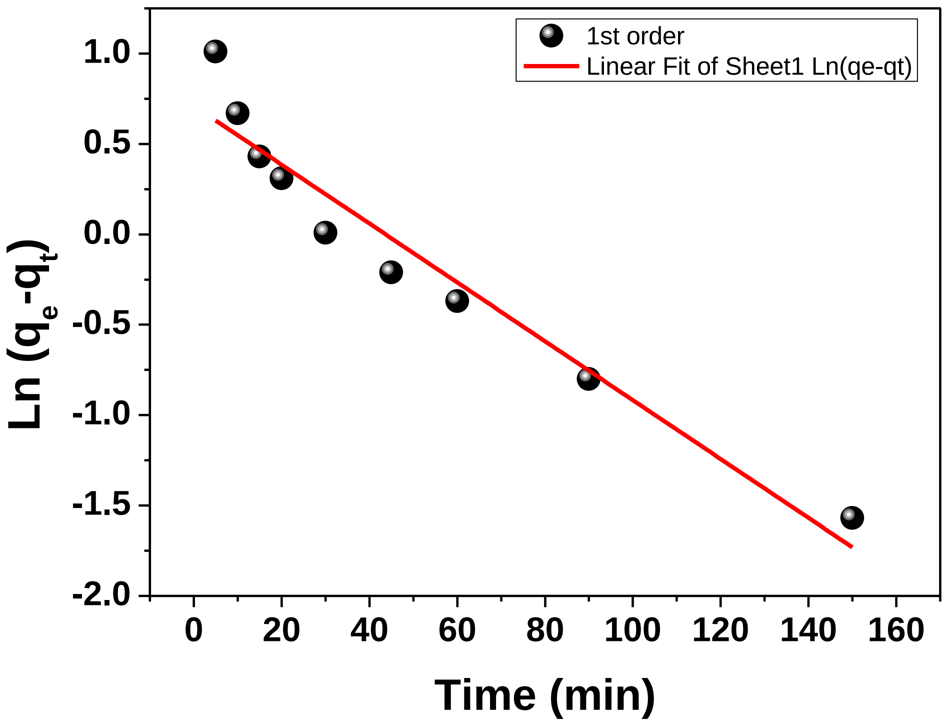

Figure S2. Linear fitting to the pseudo-first order equation, y = 0.71074 - 0.01628 x, R^2 = 0.93447

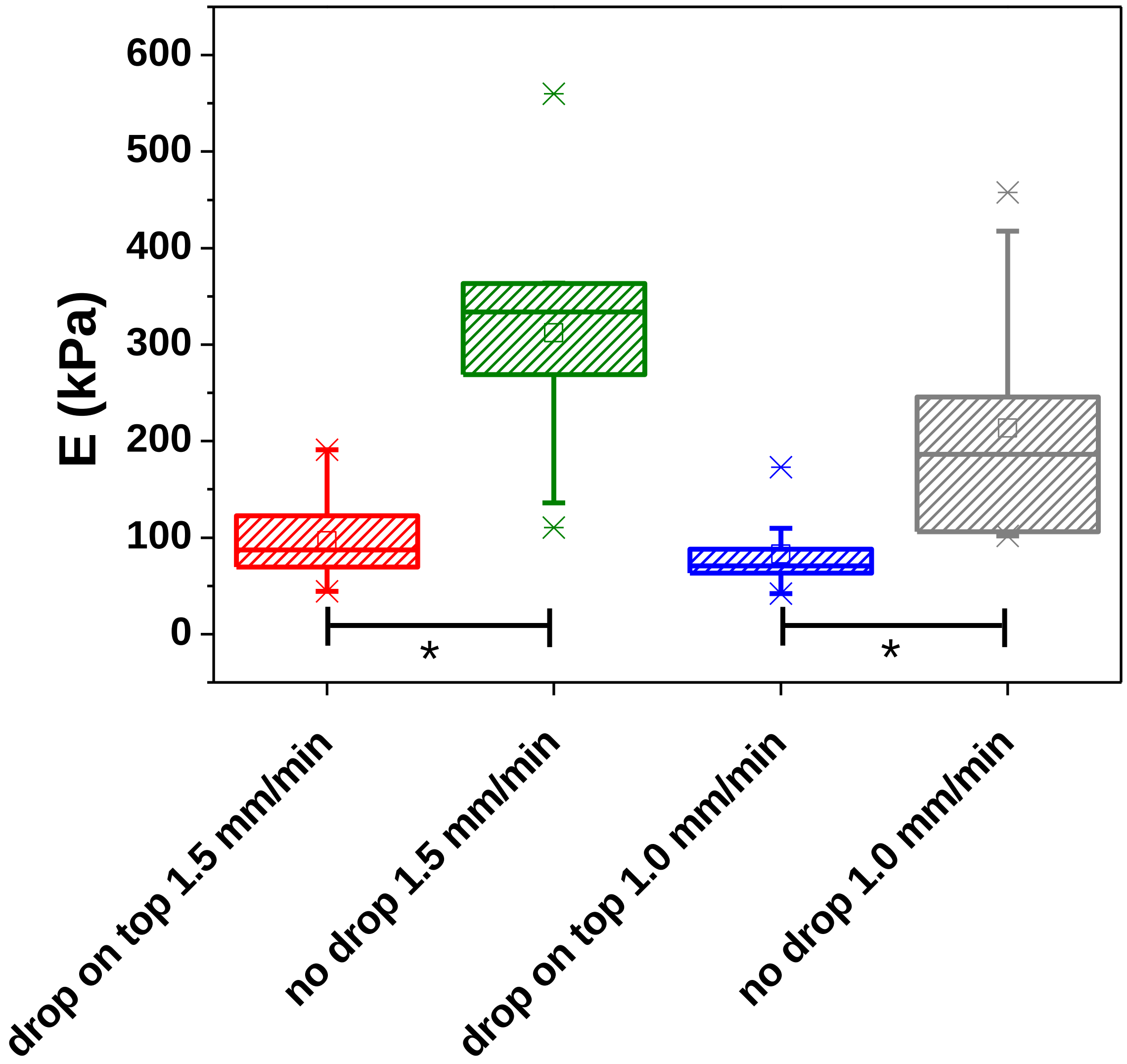

Figure S3. Distributions of E for CHI films in the swollen state obtained via tensile testing at different strain rates. Mean values (■), minimum and maximum values (–), mean 1% and 99% values (×) and median values (—). * $p < 0.05$.

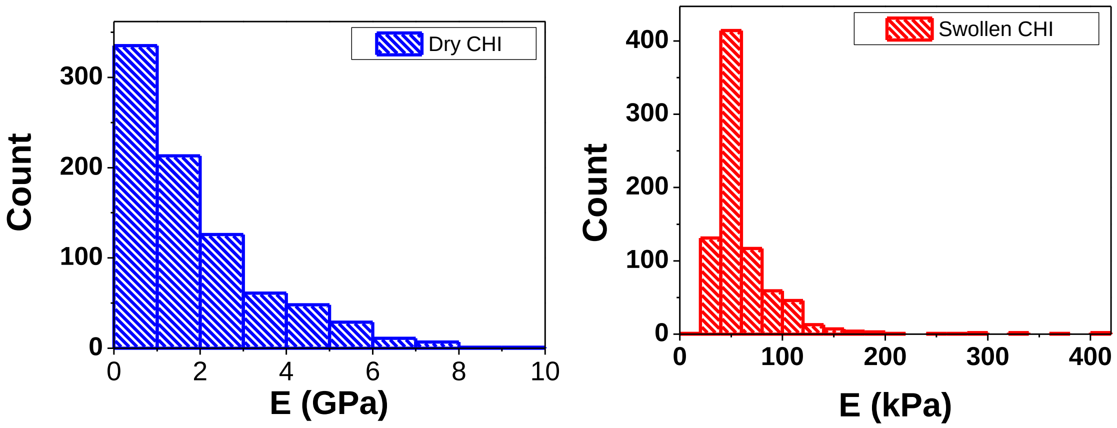

Figure S4. Histograms obtained for the indentation of CHI films. 16 x 16-pixel maps were recorded at three random regions of two samples from different batches. A total of 500 force-curves were collected for each sample.

Table S1 – Average film thickness accessed using a Digimess micrometer (0.01 mm precision) in the dry and swollen states, under different conditions.

Analysis	Tensile (dry)	Tensile (drop on top)	Tensile (no drop)
Average thickness (μm)	96 ± 11	449 ± 33	438 ± 30

Table S2 – Changes in the mass of swollen CHI films subjected to tensile testing (Sample) and not tested (Control). The time point t_0 indicates when films were removed from PBS and blotted dry. The time point t corresponds to the moment after the tensile test when the mass of both Sample and Control films was measured. Normalization factor was obtained from highest/lowest film mass ratio.

Control t_0 (g)	Control t_0 normalized	Control t (g)	Control t normalized
0.13325	0.18928	0.12755	0.18339
0.10725	0.15235	0.10050	0.14450
0.15050	0.21379	0.14275	0.20525
0.15235	0.21642	0.14450	0.20776
0.11510	0.16350	0.11145	0.16024
Sample t_0 (g)	Sample t_0 normalized	Sample t (g)	Sample t normalized
0.15940	0.27428	0.13475	0.25448
0.17465	0.30052	0.14400	0.27195
0.11705	0.20141	0.09250	0.17469
0.16370	0.28168	0.13230	0.24985
0.10150	0.17465	0.07625	0.14400
Control initial weight loss	Sample initial weight loss		
-3%	-8%		
-5%	-11%		
-4%	-15%		
-4%	-13%		
-2%	-21%		
-4% (Avg)	-14% (Avg)		
1% (Std)	5% (Std)		

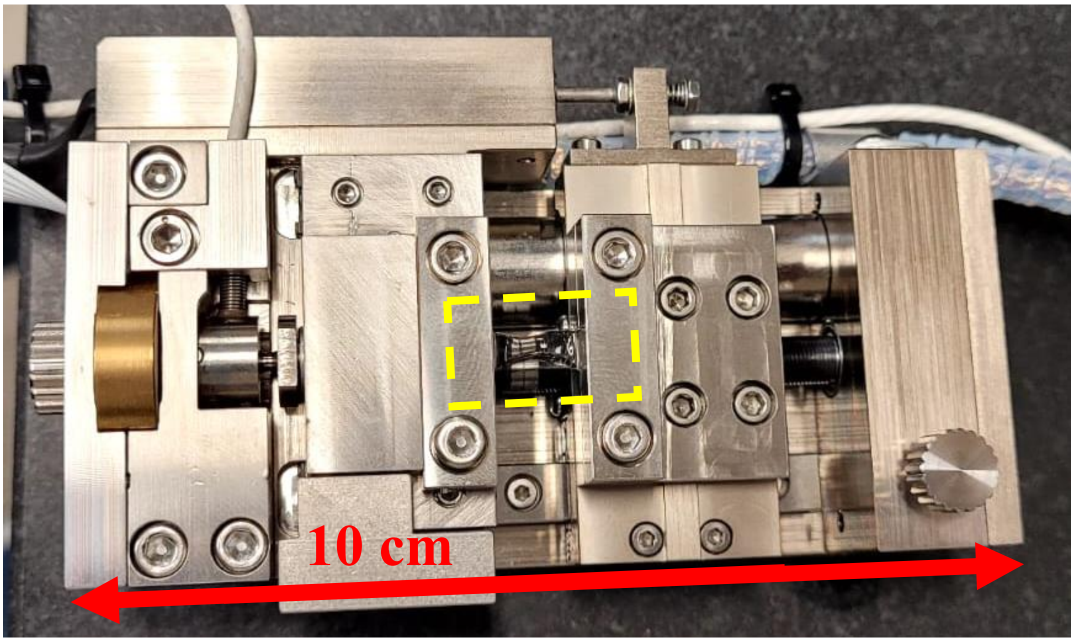

Figure S5. Swollen CHI film with buffer drop placed on top. Tensile tests were performed with a Deben® Microtest 200N, at a constant rate of 1.5 mm/min and an initial gap of 10 mm between the clamps.

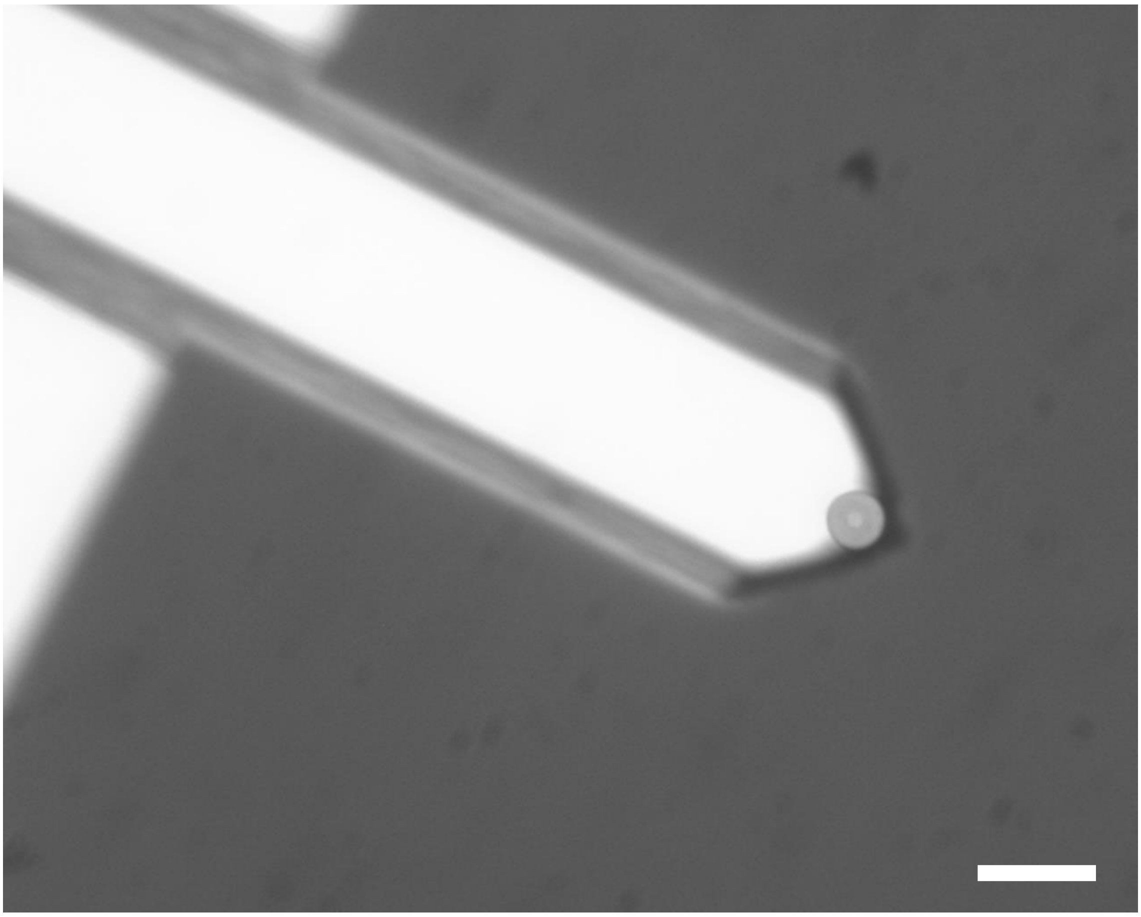

Figure S6. Optical microscopy of a colloidal silica sphere fixed with epoxy glue to the end of an 8.9 N/m tipless cantilever. The scale bar corresponds to 20 mm.

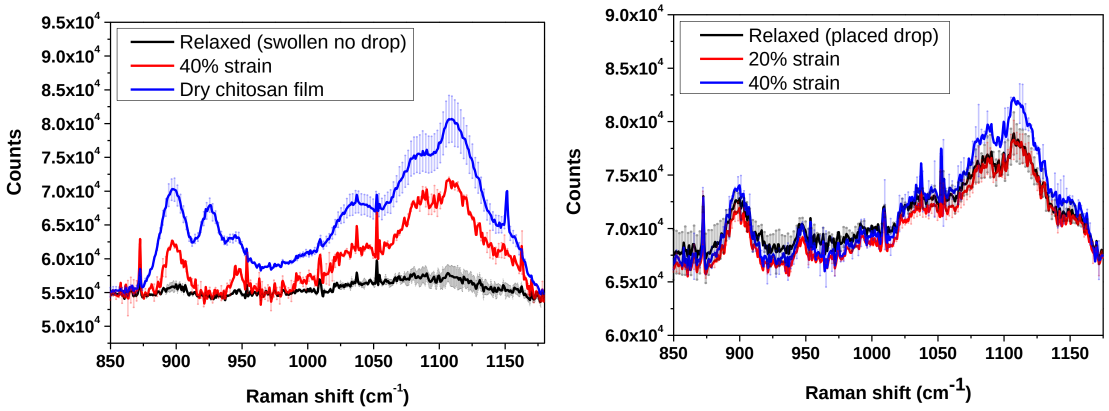

Figure S7. Raman spectra obtained for CHI films in a relaxed state and under stress: (a) Dry or swollen CHI films without PBS drop, and (b) swollen CHI films with PBS drop on the surface of the film. Spectra were averaged for n=2.
